# In Vitro Antibacterial and Antibiofilm Effects of Antiseptics and Mouthwashes on *Streptococcus mutans*

**DOI:** 10.3390/pathogens15060598

**Published:** 2026-06-02

**Authors:** Marzena Korbecka-Paczkowska, Tomasz M. Karpiński

**Affiliations:** 1 Department of Medical Microbiology, Poznań University of Medical Sciences, Rokietnicka 10, 60-806 Poznań, Poland; mkorbecka@wp.pl; 2 Medi Pharm, os. Konstytucji 3 Maja 14/2, 63-200 Jarocin, Poland

**Keywords:** dental caries, oral microbiology, biofilm thickness, antimicrobial tolerance, oral hygiene products, cariogenic bacteria

## Abstract

Background: *Streptococcus mutans* is a key etiological agent of dental caries, and its ability to form biofilms significantly increases resistance to antimicrobial agents. This study evaluated the activity of selected oral antiseptics against both planktonic and biofilm forms of *S. mutans*. Methods: Seven pure antiseptic compounds and nine commercially available mouthwashes were tested. Minimal inhibitory concentrations (MICs) were determined using a microdilution method, and their clinical relevance was assessed using the Clinical Efficiency of MIC (CEMIC) index. Antibiofilm activity was evaluated using a crystal violet assay and by measuring biofilm thickness using 3D microscopy and the Biofilm Thickness Analyzer application. Results: The highest antibacterial and antibiofilm activity was observed for octenidine (OCT), chlorhexidine (CHX), and polyhexanide (PHMB), as well as for mouthwashes containing these agents, all showing high CEMIC values. Hydrogen peroxide (H_2_O_2_) and fluoride-containing formulations also demonstrated notable activity. In contrast, ethacridine lactate and boric acid showed variable effects, while sodium hypochlorite and potassium permanganate exhibited weak antibacterial activity and no antibiofilm effect. Conclusions: OCT, CHX, and PHMB were the most effective against both planktonic and biofilm forms of *S. mutans*. H_2_O_2_ and fluoride-containing formulations also showed promising activity. These findings support the role of selected antiseptics in reducing dental plaque formation, a key factor in the development of oral diseases.

## 1. Introduction

Dental caries is one of the most prevalent chronic diseases worldwide, constituting a significant global public health and economic burden. Untreated dental caries of permanent teeth is the most common oral condition, with a global age-standardized prevalence of 27,500 (24,000–32,000) per 100,000 population [1]. It is estimated that, in 2021, there were approximately 2.24 billion cases of caries of permanent teeth and 0.52 billion cases of deciduous teeth caries worldwide [2]. Despite advances in oral hygiene practices and preventive strategies, the incidence of caries remains high, underscoring the need for more effective approaches targeting its primary etiological factors.

Dental caries is a polymicrobial disease resulting from the activity of pathobionts, commensal microorganisms that can contribute to disease under conditions of dysbiosis. A diet rich in fermentable sugars promotes ecological imbalance within the oral biofilm, favoring the proliferation of cariogenic microorganisms [3]. Among these, *Streptococcus mutans* is considered a key contributor to cariogenic biofilm development due to its exceptional ability to adhere to tooth surfaces, metabolize dietary carbohydrates, and produce organic acids that drive enamel demineralization [4]. Its capacity to damage hard dental tissues is associated with numerous virulence factors. A particularly important determinant of pathogenicity is the ability to form biofilm on the surface of tooth enamel. The *S. mutans* biofilm is a highly structured, multilayered community of bacterial cells embedded within an extracellular matrix composed of extracellular polysaccharides (EPS), extracellular DNA (eDNA), proteins, and other macromolecules [5]. EPS, present as soluble and insoluble glucans synthesized from sucrose by glucosyltransferases (GtfB, GtfC, GtfD), play a central role in biofilm development. Insoluble glucans are especially critical for bacterial adhesion and biofilm structural integrity, while also providing protection against environmental stressors such as pH fluctuations and antimicrobial agents [5,6]. Extracellular DNA, released through autolysis or active secretion, contributes to biofilm architecture by promoting cell aggregation and stabilization. Additionally, eDNA facilitates horizontal gene transfer, which may enhance bacterial adaptability and contribute to the emergence of antimicrobial tolerance [5]. Lipoteichoic acid (LTA), a surface polymer associated with the cytoplasmic membrane, is responsible for bacterial interactions and adhesion to the tooth surface. LTA also exhibits the ability to modulate the host immune response, which may contribute to chronic inflammatory conditions within the oral cavity [7].

Biofilm formation is a dynamic, multistep process that begins with initial bacterial adhesion, followed by proliferation, matrix production, and maturation into a dense and highly resistant structure [5,7]. In addition to glucan synthesis, *S. mutans* produces other enzymes relevant to cariogenesis, such as fructosyltransferase (Ftf), which synthesizes fructans that serve as a carbohydrate reservoir for the bacteria [7,8]. Glycolytic metabolism leads to the production of organic acids, primarily lactic acid, resulting in a local decrease in pH and subsequent enamel demineralization. The organism’s acidogenicity and aciduricity enable it to survive and remain metabolically active under acidic conditions, further enhancing its cariogenic potential [8,9]. The combined action of these virulence factors confers *S. mutans* with a high capacity for adhesion, persistence within the biofilm, resistance to mechanical removal, and effective destruction of dental tissues. Consequently, the formation of biofilm, referred to in the oral cavity as dental plaque, initiated by *S. mutans* over time leads to the development of common oral diseases, including dental caries, gingivitis, chronic periodontitis, and halitosis [10,11].

Current preventive strategies for dental caries rely largely on reducing the number of cariogenic bacteria, mechanical plaque removal and the adjunctive use of chemical agents such as antiseptics and mouthwashes [12,13]. These agents are particularly valuable in areas that are difficult to access with conventional oral hygiene methods like a toothbrush or dental floss. However, these approaches present several limitations, including incomplete biofilm removal, patient compliance issues, and potential adverse effects associated with long-term use [12,14]. Although *S. mutans* is generally susceptible to many antiseptics used in dentistry, studies have demonstrated that bacteria within biofilms can exhibit 10–1000-fold greater resistance to antimicrobial agents compared to their planktonic counterparts [15]. Furthermore, repeated or prolonged exposure to sublethal concentrations of antiseptics may promote adaptive responses, potentially reducing their long-term efficacy. This phenomenon has been described in other microorganisms, including *Pseudomonas aeruginosa* and *Candida albicans* [16,17]. Such adaptation raises concerns regarding decreased susceptibility, highlighting the need for systematic evaluation of antiseptic efficacy. Despite the widespread use of oral antiseptics and commercially available mouthwashes, there is limited information on their effectiveness against mature *S. mutans* biofilms. Most existing studies focus on planktonic cells, which do not accurately reflect the complex biofilm environment present in the oral cavity.

Therefore, the aim of this study was to perform a preliminary evaluation of the activity of selected oral antiseptics and commercially available mouthwashes against the cariogenic bacterium *S. mutans*, with particular emphasis on their antibiofilm efficacy. Additionally, this study seeks to assess their potential translational relevance in the prevention of dental plaque formation and caries development.

## 2. Materials and Methods

### 2.1. Streptococcus mutans Strains

Two reference strains of *Streptococcus mutans* (ATCC 35668 and ATCC 25175) were obtained from Argenta (Poznań, Poland). The strains were stored under recommended conditions and routinely subcultured prior to experiments to ensure viability and purity. Bacteria were cultured on Columbia agar supplemented with 5% sheep blood (Graso Biotech, Starogard Gdański, Poland) and incubated at 36 °C for 48 h under aerobic conditions.

### 2.2. Antiseptics

This study included an evaluation of eight individual antiseptic agents and nine commercially available mouthwashes. Among the antiseptics tested, chlorhexidine digluconate (CHX), octenidine dihydrochloride (OCT), and polyhexamethylene biguanide (PHMB) are widely recognized and routinely incorporated into oral care formulations. In contrast, sodium hypochlorite (NaOCl) is primarily applied in endodontic procedures. The remaining substances, boric acid (BA), ethacridine lactate (ET), hydrogen peroxide (H_2_O_2_), and potassium permanganate (KMnO_4_), are currently not recommended for use [18,19]. In all tests, the concentrations of the active compounds reflected those found in current commercially available oral antiseptics or topical wound care products. Additionally, 9 commercial mouthwashes with diverse compositions, containing OCT, CHX, PHMB, and fluorides, were tested. A detailed overview of the tested products, their initial concentrations of active compounds, and manufacturers is provided in Table 1 and Table 2.

### 2.3. Minimal Inhibitory Concentration (MIC)

The minimum inhibitory concentrations (MICs) of the tested antiseptics were assessed using a standard broth microdilution technique performed in sterile 96-well microplates (Nest Scientific Biotechnology, Wuxi, China). The general procedure followed previously published protocol [20], with minor modifications introduced to optimize assay conditions for *S. mutans*. Brain heart infusion (BHI) broth (Graso Biotech, Starogard Gdański, Poland) was used as the culture medium due to its suitability for the growth of fastidious oral bacteria. Each well contained a final reaction volume of 150 µL. Two-fold serial dilutions of all tested compounds were prepared starting from the initial stock concentrations specified in Table 1, ensuring a broad concentration range for accurate MIC determination. Standardized bacterial inocula were added to each well, and the microplates were incubated under aerobic conditions at 36 °C for 24 h. After incubation, MIC endpoints were determined by direct visual observation based on the absence of visible turbidity. In cases where visual assessment was ambiguous, the results were additionally verified by adding 10 µL of a 1% aqueous solution of thiazolyl blue tetrazolium bromide (MTT) (Sigma-Aldrich, Poznań, Poland), which enables metabolic activity-based differentiation between viable and inhibited bacterial growth. The lowest concentration showing no color change was recorded as the MIC value for each tested compound.

### 2.4. Clinical Efficiency of MIC (CEMIC)

To assess the clinical applicability of the MIC values obtained, the Clinical Efficiency of MIC (CEMIC) index was used. This index was determined by calculating the ratio between the minimum inhibitory concentration and the standard therapeutic concentration of the antimicrobial agent, using the following formula [17]:

$$Clinical\;Efficiency\;of\;MIC\;(CEMIC)=\frac{MIC}{Clinical\;concentration}$$

Interpretation of the results was based on the following criteria:

- CEMIC < 0.1 indicated high clinical efficiency,
- values between 0.1 and 0.9 were considered to reflect moderate efficiency,
- while values > 0.9 suggested low clinical efficiency of the compound at the tested concentration.

### 2.5. Antibiofilm Effect

Preliminary antibiofilm activity was assessed using the crystal violet assay as described previously [21]. Mature biofilms were grown in 96-well plates in BHI and incubated at 36 °C for 72 h. Biofilms were then exposed to 100 µL of antiseptics at concentrations presented in Table 1, for 5 or 60 min. Following treatment, biomass was fixed with methanol, stained with 1% crystal violet, and the dye was solubilized in 96% ethanol. Biofilms were quantified spectrophotometrically at 630 nm using a microplate reader (ELISA 250, bioMérieux, Warsaw, Poland).

Main antibiofilm activity was evaluated in digital microscopy, using 12-well culture plates (Nest Scientific Biotechnology, Jiangsu, China). Biofilms were formed by inoculating each well with 1 mL of bacterial suspension prepared in BHI broth and adjusted to a 0.5 McFarland standard. Plates were incubated at 36 °C for 48 h to allow for mature biofilm development. After incubation, wells were rinsed three times with sterile 0.9% NaCl to remove planktonic cells. Subsequently, 1 mL of each antiseptic or mouthwash solution used at its commercial concentration (as detailed in Table 1) was added to the biofilms. The contact time was 1 h at 36 °C. After exposure, residual antiseptics were removed and neutralized by treating the wells with alkaline saline peptone water (Sigma-Aldrich, Poznań, Poland) for 5 min, followed by another rinse with 0.9% NaCl. Biofilm thickness was analyzed using a Keyence VHX-S770E digital microscope (Keyence International, Mechelen, Belgium). A three-dimensional reconstruction of the biofilm surface was generated, and image data were further analyzed using an author’s Python-based application, Biofilm Thickness Analyzer [22] to calculate biofilm thickness.

### 2.6. Statistics

One-way ANOVA with Tukey post-tests was used to assess the statistical significance of differences in the biofilm thickness. All experiments were performed in triplicate. Results were considered significant at a *p*-value of less than 0.05. Data analysis was performed using InStat3 software (GraphPad Software, Boston, MA, USA).

## 3. Results

### 3.1. Minimal Inhibitory Concentration (MIC) and CEMIC

The highest antibacterial activity was demonstrated by OCT, CHX, PHMB, H_2_O_2_, as well as mouthwashes containing OCT, CHX, or PHMB. Slightly weaker activity was observed for ET and fluoride-containing mouthwashes. At the same time, all the above-mentioned products exhibited high CEMIC values. NaOCl, KMnO_4_, and BA showed the weakest MIC activity, which is also confirmed by the CEMIC index, classified as moderate for NaOCl, KMnO_4_, and BA, and as low for KMnO_4_ (Table 3 and Table 4).

### 3.2. Antibiofilm Effect

In the preliminary crystal violet assay, no statistically meaningful differences in absorbance were observed between the control and antiseptic-treated groups after 5 min of exposure (data not presented in this work). Noticeable reductions in biofilm biomass were detected after 60 min of exposure, with the exception of KMnO_4_, which did not show any measurable effect. Based on these findings, the main antibiofilm experiments were subsequently conducted using a 1 h exposure time to antiseptics and mouthwashes, as this duration provided sufficient resolution to detect treatment-related differences in biofilm structure and biomass.

In digital microscopy, the biofilm thickness for the *S. mutans* control was 453.5 ± 297.3 µm. The observed differences in the control biofilm thickness are most likely related to the use of mature biofilm formed over 72 h. At this stage, the biofilm is generally thicker and may undergo partial detachment. This can result in increased variability in measured thickness, and a wider range of values. In the MIC and CEMIC studies, the strongest antibiofilm activity was observed for OCT, CHX, and PHMB, with biofilm thickness values of 68.2 ± 77.9 µm, 24.9 ± 17.6 µm, and 72.8 ± 40.2 µm, respectively. Similarly, the strongest antibiofilm activity was shown by mouthwashes containing these compounds, as follows: Octenident (56.8 ± 39.6 µm), Octenisept Oral Mono (35.3 ± 24.2 µm), Eludril Classic (63.0 ± 18.0 µm), Implant Alfa (44.0 ± 34.8 µm), Perio Aid Intensive Care (108.9 ± 20.1 µm), and ProntOral (31.7 ± 8.1 µm). A significant antibiofilm effect was also demonstrated by H_2_O_2_ (92.5 ± 31.4 µm) and fluoride-containing mouthwashes, including Oral-B Pro Expert (196.7 ± 127.9 µm), Elmex Anti-Caries (89.6 ± 60.8 µm), and Meridol (75.2 ± 50.7 µm). Additionally, the mean biofilm reduction is presented in Table 5 as a complement to the results presented above.

**Figure 1:**
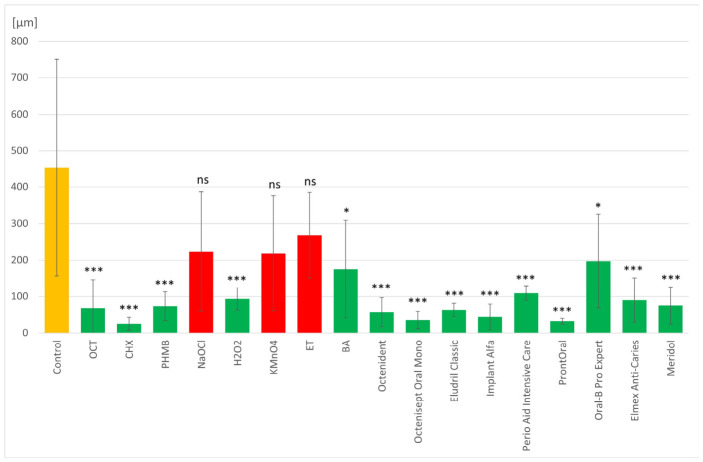
Graphical presentation of biofilm thickness after 1 h exposure to pure antiseptics and mouthwashes. Legend: ns—no statistical significance (*p* > 0.05); *—*p* < 0.05; ***—*p* < 0.001. The control was marked in yellow, active substances that significantly reduced biofilm thickness were marked in green, and compounds with no significant effect on reducing biofilm thickness were marked in red.

**Figure 2:**
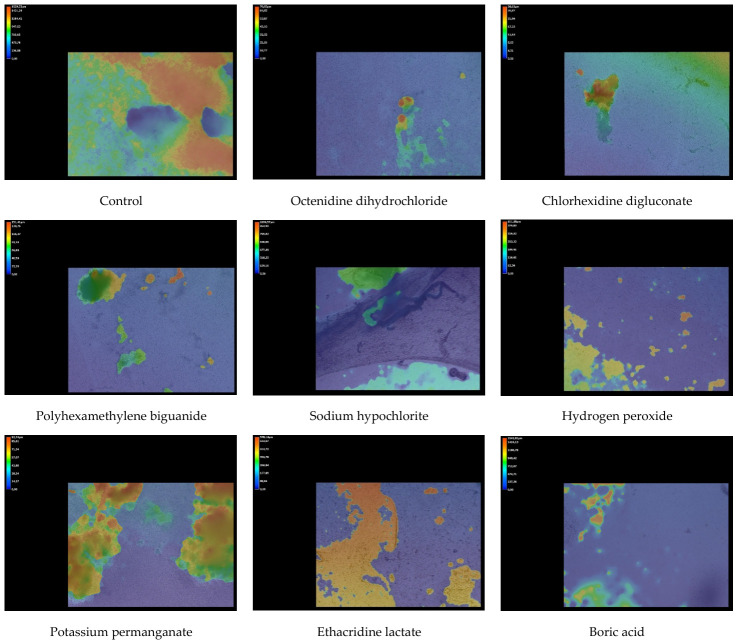
Sample images illustrating the assessment of *Streptococcus mutans* biofilm thickness following 1 h incubation with pure antiseptics. The color bar located in the upper left corner indicates biofilm thickness expressed in micrometers (µm), ranging from dark blue, representing the lowest thickness, to red, indicating the highest values. Intermediate colors correspond to increasing biofilm height. Mean biofilm thickness for each image was calculated using the Python-based Biofilm Thickness Analyzer application [22].

**Figure 3:**
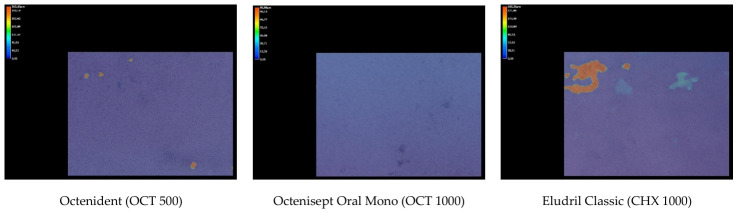

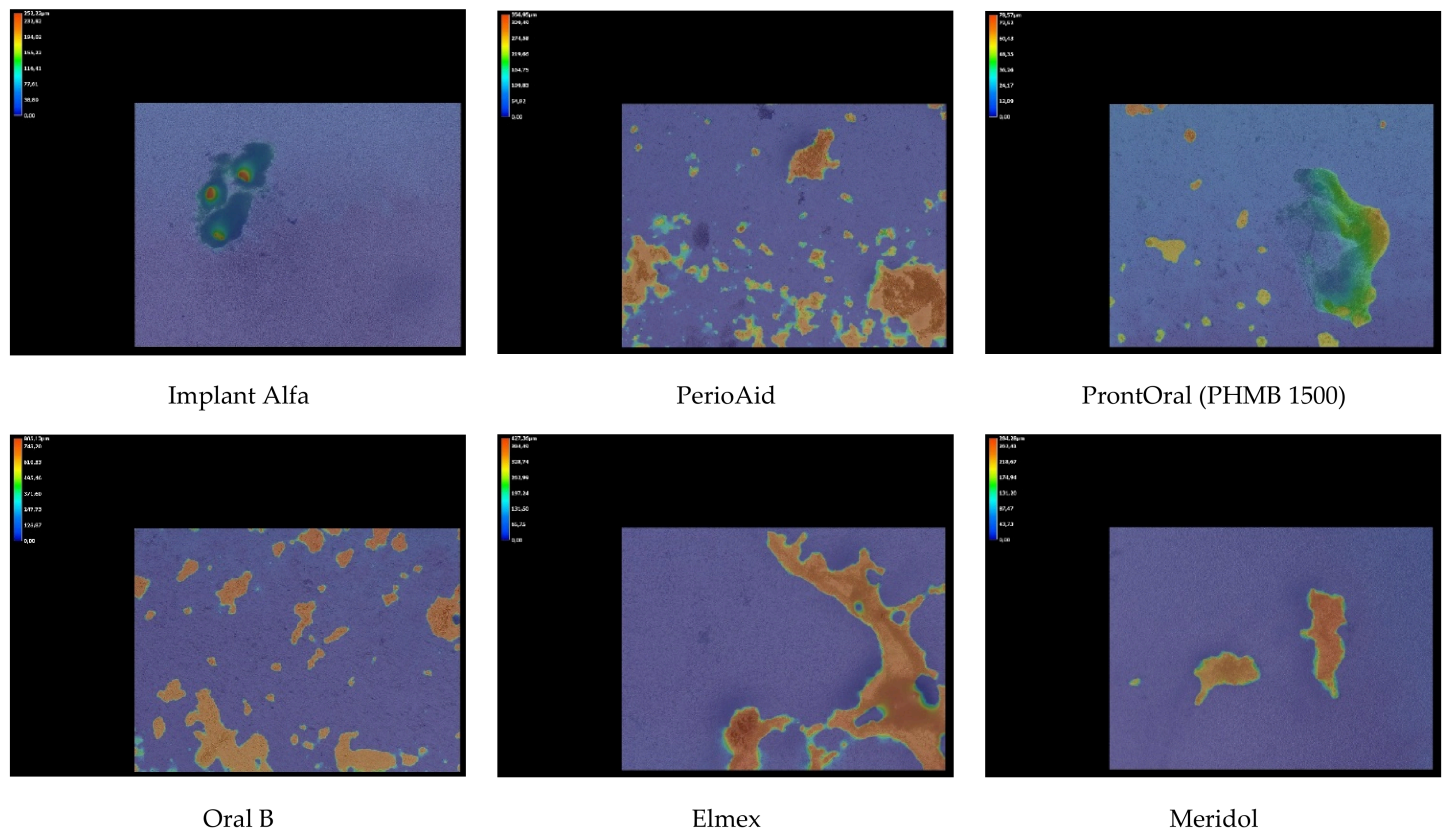
Sample images illustrating the assessment of *Streptococcus mutans* biofilm thickness following 1 h incubation with commercial mouthwashes. The description of the color interpretation is presented in Figure 2.

No antibiofilm activity was again observed for NaOCl and KMnO_4_, with values of 223.4 ± 164.3 µm and 201.5 ± 172.1 µm, respectively. It was further noted that BA exhibited antibiofilm efficacy (175.3 ± 179.3 µm), whereas ET did not (267.5 ± 116.7 µm), despite ET showing stronger activity in the MIC assays (Figure 1). Representative images of the biofilm thickness results obtained in digital microscopy are presented in Figure 2 and Figure 3.

As shown in Table 6, the highest overall activity against *S. mutans* was observed for OCT, CHX, PHMB, H_2_O_2_, and fluorides, which demonstrated both high CEMIC values and clear antibiofilm effects. These compounds were therefore identified as having the greatest potential clinical utility. In contrast, NaOCl exhibited moderate to low activity, and KMnO_4_ showed only low CEMIC values and lacked antibiofilm activity, suggesting limited applicability in the prevention of *S. mutans* biofilm formation. ET, despite high CEMIC, did not show antibiofilm activity, indicating that antibacterial effects against planktonic cells do not necessarily translate into biofilm reduction. BA showed moderate antibacterial activity and good antibiofilm effect, meaning it has partial clinical potential.

## 4. Discussion

In this study, the activity of selected antiseptics and commercial mouthwashes against *Streptococcus mutans* in both planktonic and biofilm forms was comparatively evaluated. The obtained results clearly indicate that antibacterial efficacy does not always directly correlate with antibiofilm activity, which is clinically relevant in the context of dental plaque control and caries prevention.

The strongest effects against both planktonic cells and biofilm were observed for OCT, CHX, and PHMB. These compounds, as well as mouthwashes containing them, demonstrated high CEMIC values, indicating favorable translational potential and good agreement between in vitro activity and clinically used concentrations. These findings are consistent with previous literature reports highlighting the strong bactericidal properties of these agents against *S. mutans*. Studies have shown that CHX acts against *S. mutans* at concentrations ranging from 0.25 to 1.0 to 3.1 µg/mL [23,24], which is similar to or slightly lower than those observed in our study. Some reports describe higher MIC values, for example 15.6–62.5 µg/mL for CHX [25,26,27]. Interestingly, Uzer Celik et al. reported relatively high MIC values for *S. mutans*, approximately 70 µg/mL for CHX, 120 µg/mL for OCT, and 50 µg/mL for PHMB [28]. In the biofilm experiments, we demonstrated reductions of approximately 85–92% for OCT, 76–95% for CHX, and 84–93% for PHMB. Similar findings were reported by other researchers, showing biofilm reduction in *Streptococcus* spp. of 84% for PHMB and 52% for CHX [29], while for *S. mutans* the reduction reached 81% for CHX, 96.88% for PHMB, and 74.94% for the ProntOral mouthwash [30]. An important aspect of the present study is the inclusion of antibiofilm activity assessment in a 3D model and the evaluation of biofilm thickness. It is particularly relevant that OCT, CHX, and PHMB not only inhibit bacterial growth but also effectively reduce already formed biofilm, which represents the main factor initiating dental plaque formation. Dental plaque is a structurally organized biofilm that constitutes the first stage in the development of dental caries, gingivitis, and periodontal diseases [31,32]. Therefore, the ability of antiseptics to penetrate and destabilize biofilm structures has crucial clinical significance.

The results for H_2_O_2_ and fluoride-containing formulations also indicate their relevant antibacterial and antibiofilm activity. Hydrogen peroxide, through the generation of reactive oxygen species, may exert antimicrobial activity and additionally disrupt biofilm integrity; however, its effect is dependent on environmental conditions [33,34]. MIC values for H_2_O_2_ against *S. mutans* reported in another study were 117 µg/mL and were comparable to those obtained in our work [25]. Fluoride, in addition to its classical anticaries effect associated with enamel remineralization, also inhibits bacterial metabolism. This action is based on the inhibition of several enzymes of the galactose pathway, fructose pathway, fructose/mannose-specific enzyme components in the phosphotransferase system, and lactate dehydrogenase—an enzyme involved in lactic acid synthesis [35,36]. Other studies confirm our findings and indicate that Oral-B Pro Expert mouthwash acts against *S. mutans* at dilutions of 1.56–3.125% [37,38].

ET and BA demonstrated ambiguous efficacy. Although antibacterial activity was observed under planktonic conditions, this effect did not consistently translate into biofilm reduction. Moreover, their known clinical limitations, including potential cytotoxicity and side effects, may restrict their translational potential. Regarding the literature coverage, while BA and ET have been investigated in various antimicrobial contexts, we did not identify studies specifically evaluating their activity against *S. mutans* [39,40,41,42]. We also did not find reports on directly comparable streptococcal biofilm models under the same experimental conditions in the searched databases (PubMed, Medline, and Scopus). Therefore, our work should be considered as expanding the existing evidence base for these compounds in the context of *S. mutans*.

A particularly important finding is the lack of antibiofilm activity of sodium hypochlorite (NaOCl) and potassium permanganate (KMnO_4_) under the tested conditions. Despite their well-known oxidative properties, no substantial reduction in *S. mutans* biofilm was observed. This observation may be related to limited penetration into the biofilm structure and/or its rapid neutralization in the presence of organic matter. Consequently, their application in dental plaque prevention appears limited. It is also important to note that the antimicrobial activity of NaOCl reported in the literature is concentration-dependent, with effects observed at relatively high levels (e.g., 6560 µg/mL and even up to 30,000 µg/mL, corresponding to approximately 3%) [43,44]. At the same time, cytotoxic concentrations of NaOCl for human cells range from 0.05% to 0.5% [45,46]. This indicates a narrow therapeutic window and suggests that in certain situations, effective antibacterial concentrations may approach levels associated with tissue toxicity, including tissue damage and necrosis [47,48]. Therefore, while NaOCl remains used mainly in endodontics, its activity should be interpreted in the context of concentration, exposure conditions, and limitations of in vitro biofilm models rather than as an absolute indicator of clinical efficacy.

Although BA, ET, H_2_O_2_, and KMnO_4_ are not currently recommended in modern clinical guidelines, mainly due to side effects, cytotoxicity or insufficient activity [49,50,51,52], they are still used in some regions of the world, including Europe, particularly by patients on their own initiative, without medical supervision, both for wound care and as mouth rinses (verbal information). Moreover, H_2_O_2_ is still occasionally used for oral rinsing, while KMnO_4_ was commonly applied in dental procedures such as scaling or curettage until relatively recently, and may still be used by some practitioners [52,53]. The added value of including these substances lies in several aspects. First, it allows assessment of antimicrobial activity of compounds that may still be encountered in clinical or self-treatment settings. Second, there is a relatively limited number of recent studies evaluating their in vitro efficacy under comparable experimental conditions. Third, their inclusion provides the opportunity to compare commonly used antiseptics with historically used agents, helping to better position their antimicrobial activity.

In the context of evaluating antibiofilm activity, it is important to consider results obtained after as little as 5 min of exposure, as they better reflect real clinical conditions. This is particularly relevant for preparations used in the oral cavity, where the contact time with the biofilm is very limited; in the case of mouthwashes, it typically ranges from only 10 to 30 s [54,55]. If no significant biofilm reduction is observed within such a short time, the limited antibiofilm efficacy in vivo should be clearly emphasized in the final part of the discussion. Additionally, it should be taken into account that biofilms occurring under natural conditions are usually multispecies, which increases their tolerance to antimicrobial agents. Consequently, the actual antibiofilm activity of the tested substances may be even lower than that observed in in vitro models based on single microbial species.

A limitation of this study is the lack of assessment of biofilm viability, as only thickness was analyzed. Future studies should include live/dead cell assays to provide a more comprehensive evaluation of antibiofilm activity. The next limitation is in vitro nature of this study and the use of a single-species biofilm model, which does not account for the complex interactions occurring in the oral environment, such as the presence of saliva, a multispecies microbiome, and fluid dynamics. Nevertheless, the application of a 3D biofilm model and the evaluation of biofilm thickness provide a closer approximation to clinical conditions and increase the scientific value of the obtained findings.

Additionally, the study was performed using two reference strains of *S. mutans*, which does not reflect the substantial phenotypic and genotypic variability of clinical isolates, including differences in biofilm-forming ability and tolerance to antimicrobial agents. Therefore, the results may not fully represent responses of strains isolated from patients. However, many studies indicate that the activity and MIC values of individual antiseptics are generally comparable within the same species [16,17,42,56,57].

From a translational perspective, the obtained results indicate that effective control of *S. mutans* and dental plaque requires the use of formulations with a dual mechanism of action, combining antibacterial and antibiofilm effects. However, the exposure times used in this study were longer than the actual contact time of mouthwashes with oral surfaces during routine hygiene. The findings suggest that, particularly under in vivo conditions, short-term retention of formulations in the oral cavity may not exert a meaningful antibiofilm effect, but rather primarily reduce the number of planktonic microorganisms.

## 5. Conclusions

Based on the conducted studies, the following conclusions can be drawn:

1. Octenidine, chlorhexidine, polyhexanide, hydrogen peroxide, and fluoride-containing formulations showed the strongest antibacterial and antibiofilm activity against *S. mutans*, indicating their high potential for disruption of biofilm formation.
2. Sodium hypochlorite and potassium permanganate demonstrated weak antibacterial effects and no measurable antibiofilm activity, suggesting limited effectiveness in preventing *S. mutans* biofilm development.
3. Ethacridine lactate and boric acid showed limited or inconsistent antibacterial efficacy. Additionally, their potential adverse effects and safety concerns further limit their suitability for routine use in dentistry.
4. The results highlight that effective control of *S. mutans* requires agents with both antibacterial and antibiofilm properties, as biofilm formation plays a key role in dental plaque development and early caries progression.

## Figures and Tables

**Table 1:** Pure antiseptics used in the study.

Name of the Antiseptic	Abbreviation	Initial Concentration [µg/mL]	Manufacturer
Octenidine dihydrochloride	OCT	500	Schülke & Mayr GmbH, Norderstedt, Germany
Chlorhexidine digluconate	CHX	1000	Sigma-Aldrich, Poznań, Poland
Polyhexamethylene biguanide; Polihexanide	PHMB	1000	Arxada AG, Basel, Switzerland
Sodium hypochlorite	NaOCl	100	Cerkamed, Stalowa Wola, Poland
Hydrogen peroxide	H_2_O_2_	30,000	Hasco-Lek S.A., Wrocław, Poland
Potassium permanganate	KMnO_4_	10,000	Hasco-Lek S.A., Wrocław, Poland
Ethacridine lactate	ET	1000	Herbapol, Poznań, Poland
Boric acid	BA	30,000	Herbapol, Poznań, Poland

**Table 2:** Commercial mouthwashes used in the study.

Mouthwash	Active Compound/s and Concentrations [µg/mL]	Excipients	Manufacturer
Octenident^®^	OCT 800	aqua, PEG-40 hydrogenated castor oil, phenoxyethanol, glycerin, aroma, sodium gluconate, sucralose, citric acid, BHT	Schülke & Mayr GmbH, Norderstedt, Germany
Octenisept Oral Mono^®^	OCT 1000	glycerol, sodium gluconate, citric acid, disodium phosphate dihydrate, macrogolglycerol hydroxystearate, sucralose, purified water, mint flavor	Schülke & Mayr GmbH, Norderstedt, Germany
Eludril Classic^®^	CHX 1000	glycerin, alcohol, water, aroma, CI 16255, chlorobutanol, diethylhexyl sulfosuccinate, limonene, menthol	Pierre Fabre, Cahors, France
Implant Alfa	CHX 2000	aqua, glycerin, *Linum usitatissium* seed extract, *Salvia officinalis* extract, *Chamomilla recutita* flower extract, *Arnica montana* flower extract, *Quercus robur* bark extract, *Rosmarinus officinalis* leaf extract, propylene glycol, panthenol, PEG-40 hydrogenated castor oil, xylitol, aroma, allantoin, sodium saccharin	Atos M.M., Warsaw, Poland
Perio Aid Intensive Care^®^	CHX 1200; cetylpyridinium chloride (CPC) 500	aqua, glycerin, propylene glycol, xylitol, PEG-40 hydrogenated castor oil, methylparaben, potassium acesulfame, sodium saccharin, neohesperidin dichalcohe, aroma, C.I. 42090	Dentaid, Barcelona, Spain
ProntOral^®^	PHMB 1500	water, PEG-40 hydrogenated castor oil, fragrance, sodium cyclamate, undecylenamide dopropyl betaine	B Braun, Melsungen, Germany
Oral-B Pro Expert	Sodium fluoride 500; CPC	water, glycerin, polysorbate 20, aroma, methylparaben, sodium saccharin, sodium benzoate, propylparaben, Cl 42,051, Cl47005	Procter & Gamble, Schwalbach, Germany
Elmex Anti-Caries^®^	Sodium fluoride, Olaflur (total fluorides 250); PHMB	water, PEG-40 hydrogenated castor oil, aroma, potassium acesulfame, hydrochloric acid	Colgate Palmolive, New York, NY, USA
Meridol^®^	Stannous fluoride, Olaflur (total fluorides)	water, xylitol, PVP, PEG-40 hydrogenated castor oil, aroma, sodium saccharin, CI 42,051	Colgate Palmolive, New York, NY, USA

**Table 3:** Minimal inhibitory concentrations (MIC) and Clinical Efficiency of MIC (CEMIC) of the tested antiseptic compounds against *S. mutans*.

Antiseptic and Initial Concentration of the Active Compound [µg/mL]	MIC		CEMIC	
	As Product Concentration [%]	As Active Antiseptic Concentration [µg/mL]	Values	Clinical Efficiency
Octenidine dihydrochloride; 500	0.195	0.975	0.00195	High
Chlorhexidine digluconate; 1000	0.098–0.195	0.98–1.95	0.00098–0.00195	High
Polyaminopropyl biguanide; 1000	0.39–0.78	3.9–7.8	0.0039–0.0078	High
Sodium hypochlorite; 100	50–100	50–100	0.5–1.0	Moderate-Low
Hydrogen peroxide; 30,000	0.39–0.78	117–234	0.0039–0.0078	High
Potassium permanganate; 10,000	100	10,000	1.0	Low
Ethacridine lactate; 1000	3.125–6.25	31.25–62.5	0.0313–0.0625	High
Boric acid; 30,000	12.5	3750	0.125	Moderate

Interpretation of the CEMIC results: <0.1 means high clinical efficiency; 0.1–0.9 indicates moderate efficiency, and >0.9 means low clinical efficiency of the compound at the tested concentration [17].

**Table 4:** Minimal inhibitory concentrations (MIC) and Clinical Efficiency of MIC (CEMIC) of the tested mouthwashes against *S. mutans*.

Mouthwash and Active Compound/s Concentration [µg/mL]	MIC		CEMIC	
	As Product Concentration [%]	As Active Antiseptic Concentration [µg/mL]	Values	Clinical Efficiency
Octenident^®^; OCT 800	0.098	0.784	0.00098	High
Octenisept Oral Mono^®^; OCT 1000	0.049	0.49	0.00049	High
Eludril Classic^®^; CHX 1000	0.098–0.195	0.98–1.95	0.00098–0.00195	High
Implant Alfa^®^; CHX 2000	0.098	1.95	0.00098	High
Perio Aid Intensive Care^®^; CHX 1200 + CPC 500	0.098–0.195	1.176–2.34 + 0.49–0.975	0.00098–0.00195	High
ProntOral^®^; PHMB 1500	0.39	5.85	0.0039	High
Oral-B Pro Expert^®^; fluorides 500	3.125	15.6	0.0313	High
Elmex Anti-Caries^®^; fluorides 250	6.25	15.6	0.0625	High
Meridol^®^; fluorides 250	6.25	15.6	0.0625	High

Interpretation of the CEMIC results: <0.1 means high clinical efficiency; 0.1–0.9 indicates moderate efficiency, and >0.9 means low clinical efficiency of the compound at the tested concentration [17].

**Table 5:** Range of average reduction in *Streptococcus mutans* mature biofilm following 1 h treatment with antiseptics and mouthwashes.

Pure Compounds or Mouthwashes with Below Antiseptic	Biofilm Reduction [%]
Octenidine dihydrochloride	85–92.2
Chlorhexidine digluconate	76.0–94.5
Polyaminopropyl biguanide	83.9–93.0
Sodium hypochlorite	50.7
Hydrogen peroxide	79.6
Potassium permanganate	51.9
Ethacridine lactate	41.0
Boric acid	61.3
Fluorides	56.6–83.4

**Table 6:** Summary of the antiseptics and mouthwashes activity against *Streptococcus mutans.*

Pure Compounds or Mouthwashes with Below Antiseptic	Clinical Efficiency of MIC (CEMIC)	Antibiofilm Activity	Potential Clinical Relevance Against *S. mutans*
Octenidine dihydrochloride	High	Yes	Yes
Chlorhexidine digluconate	High	Yes	Yes
Polyaminopropyl biguanide	High	Yes	Yes
Sodium hypochlorite	Moderate-Low	No	**No**
Hydrogen peroxide	High	Yes	Yes
Potassium permanganate	Low	No	**No**
Ethacridine lactate	High	No	**No**
Boric acid	Moderate	Yes	Partial
Fluorides	High	Yes	Yes

## Data Availability

The original contributions presented in the study are included in the article, further inquiries can be directed to the corresponding author.
